# Methods for Three-Dimensional All-Optical Manipulation of Neural Circuits

**DOI:** 10.3389/fncel.2018.00469

**Published:** 2018-12-17

**Authors:** Emiliano Ronzitti, Valentina Emiliani, Eirini Papagiakoumou

**Affiliations:** Wavefront Engineering Microscopy Group, Photonics Department, Institut de la Vision, Sorbonne Université, Inserm S968, CNRS UMR7210, Paris, France

**Keywords:** light-shaping, three-dimensional photostimulation, three-dimensional functional imaging, all-optical neuronal studies, optogenetics, neural circuits

## Abstract

Optical means for modulating and monitoring neuronal activity, have provided substantial insights to neurophysiology and toward our understanding of how the brain works. Optogenetic actuators, calcium or voltage imaging probes and other molecular tools, combined with advanced microscopies have allowed an “all-optical” readout and modulation of neural circuits. Completion of this remarkable work is evolving toward a three-dimensional (3D) manipulation of neural ensembles at a high spatiotemporal resolution. Recently, original optical methods have been proposed for both activating and monitoring neurons in a 3D space, mainly through optogenetic compounds. Here, we review these methods and anticipate possible combinations among them.

## Introduction

Controlling and monitoring neuronal activity with a light has become a common practice in many neurobiological studies, throughout the last decade. The continuously expanding toolbox of molecular probes that activate/inhibit (Herlitze and Landmesser, 2007; Airan et al., 2009; Levitz et al., 2013; Klapoetke et al., 2014; Shemesh et al., 2017; Becker et al., 2018; Guruge et al., 2018; Mardinly et al., 2018) or image (Emiliani et al., 2015; Kim et al., 2018) neuronal activity as well as the development of original light-microscopy methods for stimulating these tools (Ronzitti et al., 2017b; Chen et al., 2018c; Yang and Yuste, 2018), has tremendously contributed to the direction of research and has led to innovative experimental concepts (Rickgauer et al., 2014; Carrillo-reid et al., 2016). Photostimulation via optogenetics and/or uncaging (Kwon et al., 2017) is suitable for single cell and, most importantly, circuit studies, since light gives access to a large number of targets simultaneously, at high spatial precision via parallel illumination methods (Papagiakoumou et al., 2010; Packer et al., 2015; Forli et al., 2018; Yang et al., 2018).

Circuit studies are usually performed *in vivo* by light-stimulation at near-infrared to minimize scattering effects and optimize spatial resolution via non-linear multiphoton absorption processes. Ideally, these studies also demand three-dimensional (3D) accessibility both for activation and imaging at physiological time scales (few-ms scale activation and imaging). 3D imaging approaches enable the use of complementary strategies to access volumes extending up to a few hundred μms in the axial direction, by proposing the use of piezo scanners to scan the objectives in specific trajectories (Göbel et al., 2007), acousto-optic deflectors (Reddy et al., 2008; Grewe et al., 2010; Katona et al., 2012; Nadella et al., 2016), tunable lenses (Grewe et al., 2011; Fahrbach et al., 2013; Kong et al., 2015), spatiotemporal multiplexing (Cheng et al., 2011; Ducros et al., 2013), light field microscopy (Prevedel et al., 2014), or Bessel beam excitation (Lu et al., 2017) and reaching tens of Hz imaging frequencies, of neuronal activity *in vivo* (Göbel et al., 2007; Grewe et al., 2011; Katona et al., 2012; Nadella et al., 2016). 3D functional imaging of neurons has more recently been demonstrated at even larger volumes, reaching 0.5 mm in *z*, using a temporally focused Gaussian beam excitation at the size of neuron soma (Prevedel et al., 2016), with large field views up to 5 mm in *xy* (Sofroniew et al., 2016; Stirman et al., 2016) or in two different areas of the brain (Lecoq et al., 2014; Chen et al., 2016).

**FIGURE 1:**
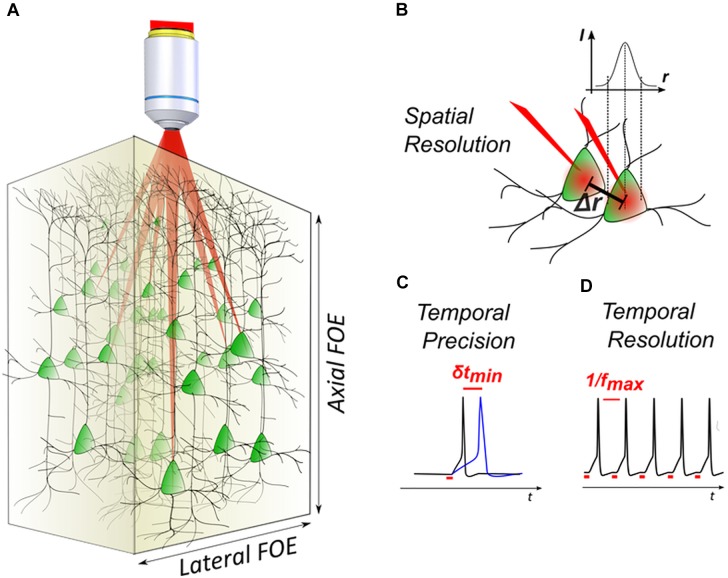
3D light-targeted photostimulation. **(A)** SLM-based multiplexing strategies allow to target opsin-expressing neurons over axial and lateral fields of excitation, extending over a few hundred microns in the brain. **(B)** The photostimulation resolution defines the minimal distance *Δr* between two targets, at which each target can be individually activated. **(C)** Photostimulation temporal performances are linked to the photostimulation temporal precision, that is the timing precision *δt* in evoking action potentials (APs) with repetitive stimulations (i.e., light-evoked spike jitter) and **(D)** the temporal resolution, that is the minimal time interval between two consecutive light-evoked APs (i.e., the maximum light-driven neuronal firing rate *f*_max_).

The development of 3D photoactivation methods is more recent. These systems are based on the use of Computer-Generated Holography (CGH) (Papagiakoumou et al., 2018; Yang and Yuste, 2018). Although this technique was established for the projection of 3D patterns (Piestun et al., 1996; Haist et al., 1997) or diffraction-limited spots (Liesener et al., 2000) via spatial light modulators (SLMs) several years ago, its use in neuroscience for simultaneous activation of multiple targets in two (Lutz et al., 2008; Nikolenko et al., 2008; Dal Maschio et al., 2010) or three dimensions (Yang et al., 2011; Go et al., 2012; Hernandez et al., 2016; Dal Maschio et al., 2017) was established only during the last decade. Thanks to 3D-CGH, used either solely (parallel methods) or in its diffraction-limit version in combination with scanning of the holographic beamlets (hybrid methods) [see (Papagiakoumou, 2013; Ronzitti et al., 2017b) for detailed description of these approaches], it is possible nowadays to activate multiple neurons providing both the adequate temporal resolution, as well as the spatial resolution for single-cell precision (Figures 1A,B).

With regards to temporal resolution, it is helpful to define the notion of *temporal precision* in optogenetic activation, i.e., the degree of reproducibility of the occurrence timing of a photo-evoked AP (also indicated as photo-evoked spike jitter) (Figure 1C) and *temporal resolution*, i.e., the time needed to photo-evoke an AP, ultimately linked to the maximum achievable light-driven neuronal firing rate (Figure 1D). Minimizing those two parameters helps to reproduce precise temporal patterns of activity that in combination with multicell activation, enables to mimic the physiological activity of a network. It has been shown that parallel photoactivation methods (Ronzitti et al., 2017b) can easily achieve short timescales during optogenetic activation (few-ms temporal resolution and sub-ms jitter) (Chaigneau et al., 2016; Ronzitti et al., 2017a; Shemesh et al., 2017; Chen et al., 2018b). Later studies using scanning methods, have also shown a high temporal specificity, reaching a millisecond jitter by using high power in the excitation spots (Yang et al., 2018).

With regards to 3D spatial resolution, scanning methods using 3D-CGH show an intrinsically good spatial resolution, thanks to the small spot size of the excitation beam (close to diffraction limit). Nevertheless, resolution can dramatically decrease when using intensities close to the saturation levels for the opsin, which results in out-of-focus excitation (Rickgauer and Tank, 2009; Andrasfalvy et al., 2010). Parallel methods use illumination shapes that cover the whole cell body, in order to achieve parallel recruitment of all opsins on the cell membrane to improve efficiency (Papagiakoumou et al., 2010). This, however, causes a quick deterioration of the axial resolution, which scales linearly or quadratically with lateral size, for holographic or Gaussian beams, respectively (Oron et al., 2012). Parallel methods can provide a good axial resolution when combined with temporal focusing (TF) (Papagiakoumou et al., 2008, 2010; Oron et al., 2012; Rickgauer et al., 2014). Notably, temporally focused light-shaping methods allow to preserve sharp borders of the excitation pattern (Papagiakoumou et al., 2010), even through scattering media (Bègue et al., 2013; Papagiakoumou et al., 2013). However, because TF works by dispersing the spectral frequencies of a femtosecond light pulse at a specific plane (Oron et al., 2005), special configurations are needed to extend the methods in 3D.

Here we review the methods proposed so far for 3D photoactivation and present the possibilities for combination with 3D imaging modalities, to establish precise and flexible microscopy methods for all-optical manipulation of neural circuits. The methods we present here have mostly been developed for optogenetic photostimulation but in principle, any other photoactivation technique, such as uncaging of caged neurotransmitters, or activation of photoactivable proteins, could benefit from them.

## All-Optical Manipulation

The very first experiment of all-optical manipulation of neurons was demonstrated by activating cells in neocortical slices via two-photon (2P) uncaging of MNI-glutamate with multiple beamlets generated with a diffractive optical element, or one single beam multiplexed in time, and 2P Ca^2+^ imaging (Nikolenko et al., 2007). Similarly, a few years later in two other papers researchers measured Ca^2+^ signals in neurons while uncaging MNI-glutamate (Dal Maschio et al., 2010; Anselmi et al., 2011). In (Dal Maschio et al., 2010) the optical system incorporated 2P 2D-CGH in the optical path of a commercial 2P scanning microscope and it could exchange holographic or scanning stimulation between the uncaging and imaging beams. In (Anselmi et al., 2011) 3D-CGH with diffraction-limited spots was combined with a remote-focusing system (Botcherby et al., 2007, 2012) to perform functional imaging along tilted dendrites of hippocampal pyramidal neurons in brain slices. Although the first demonstrations of combined activation and imaging of neurons used uncaging, the term *all-optical* is mostly related to the combination of functional imaging and optogenetic activation. In 2014, a milestone was achieved when an important number of scientific studies showing the activation of neurons *in vivo* in rodents via optogenetic molecules and the imaging of Ca^2+^ responses with GCaMP, took place (Vogt, 2015), using either visible (Szabo et al., 2014) or 2P light stimulation (Rickgauer et al., 2014; Packer et al., 2015). More publications followed, using 2D (Carroll et al., 2015; Carrillo-reid et al., 2016; Bovetti et al., 2017; Förster et al., 2017; Forli et al., 2018), and more recently 3D stimulation (Hernandez et al., 2016; Dal Maschio et al., 2017; Mardinly et al., 2018; Yang et al., 2018).

Despite these very important studies, full optical neuronal control remains a challenge in terms of achieving reliable delivery and expression of sensors and actuators in the same neurons, eliminating the cross-talk between imaging and activation, and recording and stimulating with a single-neuron and a single-action-potential precision (Emiliani et al., 2015). These problems have been discussed exhaustively in other recent reviews (Emiliani et al., 2015; Ronzitti et al., 2017b; Chen et al., 2018c). Here we will focus on reviewing the recent developments for 3D all-optical manipulation.

### 3D Photoactivation

#### Fully Parallel Methods

Fully parallel optical methods proposed for 3D activation in an all-optical configuration have been presented for optogenetics and make use of extended light-pattern formation to cover the entire neuron soma. Light-patterns can be either large Gaussian beams generated by underfilling the objective numerical aperture (NA; low-NA Gaussian beams), or beams created with more flexible light-patterning methods such as CGH, generalized phase contrast (GPC) or amplitude modulation. As already mentioned, the limit of extended light patterns is the deterioration of the axial confinement, an issue that can be solved by using temporal focusing. Common experimental configurations of TF make use of a diffraction grating in a plane conjugate to the focal plane of the microscope objective (image formation plane), separating the spectral frequencies of the laser femtosecond pulses (dispersion of different wavelengths in different angles) (Oron et al., 2005; Zhu et al., 2005). In other words, the image projected at the grating plane is the image formed at the sample plane, while 2P absorption of light projected in any other plane before or after the grating, as it is the case when you generate a 3D pattern distribution, will be strongly weakened by the pulse broadening. This has, until recently, limited TF-light shaping to 2D configurations. Methods have been proposed for axial displacement of the TF plane for Gaussian beams, by introducing group velocity dispersion (Durst et al., 2006, 2008; Dana and Shoham, 2012). However, they are only suitable for remotely displacing one plane and they are not compatible with light-patterning techniques (Leshem et al., 2014), as they can displace the TF plane but not the spatial focusing one.

Hernandez et al. (2016) solved the problem, by introducing the axial displacement mechanism after the grating for TF, which decoupled lateral light shaping from axial displacements. The system used a conventional 2D-CGH with TF for lateral light-patterning and a second SLM placed at a Fourier plane after the grating to introduce the desired axial shift from the original focal plane, via a lens-effect phase modulation. This configuration also enabled the generation of different excitation patterns at distinct axial planes, by addressing the two SLMs in multiple regions, tiled vertically to the direction of dispersion for TF. With this configuration, researchers demonstrated for the first time the generation of multi-plane temporally focused patterns, reaching a volume of 240 × 240 × 260 μm^3^ with the axial confinement varying from 5 μm Full Width Half Maximum (FWHM) at the center of the field of excitation (FOE) to 10 μm at the edges of it, tested with spots of 20 μm in diameter. The number of regions equal to the number of planes to be addressed. The system was used for selective 2P 3D photoconversion of the Kaede protein (Isobe et al., 2010) in the brain of zebrafish larvae (photoconversion at 800 nm with 0.1–4.0 mW/μm^2^ depending the illumination duration) and for 2D optogenetic activation of ChR2 in zebrafish spinal cord neurons co-expressing GCaMP5G (excitation at 900 nm with 0.6 mW/μm^2^). Monitoring of Ca^2+^ traces in that case was performed with visible illumination and two-color HiLo imaging (Lim et al., 2008) (at ∼30.8 μW/mm^2^).

**FIGURE 2:**
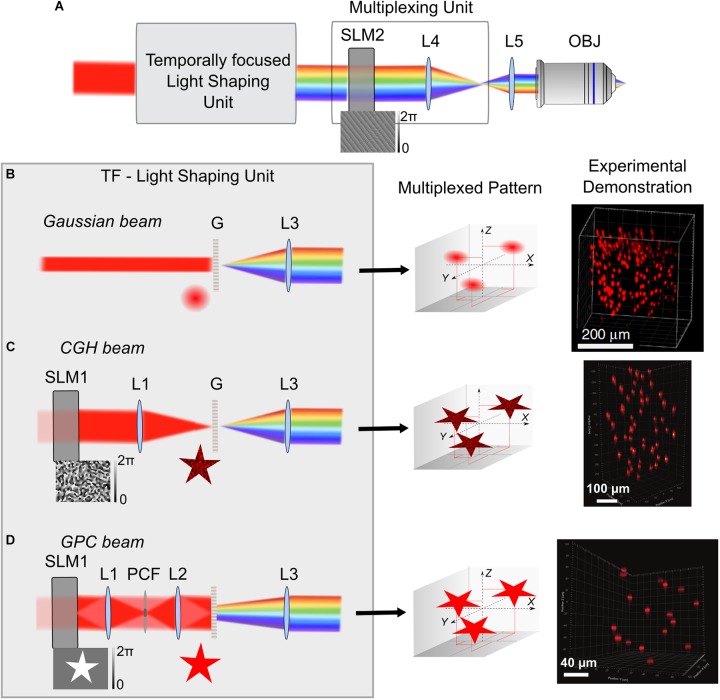
3D multiplexed temporally focused light shaping. **(A)** Optical systems for 3D temporally focused light shaping consist of a light-shaping unit for creating a 2D temporally focused pattern and a multiplexing unit, using a SLM (SLM2) at a Fourier plane of the TF system, to replicate the 2D pattern in several positions in 3D via 3D-CGH (example of a phase profile for projecting 3 diffraction-limited spots in different positions is shown on the bottom of SLM2). After the light-shaping unit, the beam is represented with its spectral frequencies diffracted, because of spectral diffraction by the grating G. After phase modulation on SLM2 the beam is imaged by two lenses (here L4 and L5) to the back aperture of the microscope objective. **(B–D)** Different cases for the TF-light-shaping unit: **(B)** Gaussian beam. In this case the grating is illuminated with a collimated Gaussian beam of a suitable size, usually adjusted with a telescope of lenses (not shown here). Configuration with a non-collimated beam (by introducing a lens prior to the grating) was used by Pégard et al. (2017) in order to increase the illuminated area of SLM2. Middle. Illustration of the example for projection of 3 Gaussian replicas, when the SLM2 is illuminated with the phase profile shown in **A**. Right. Experimental demonstration showing projection of 200 Gaussian beams in a 350 × 350 × 280 μm^3^ volume, adapted from Pégard et al. (2017). **(C)** CGH beam. A SLM (SLM1) and a lens (L1) are used for holographic pattern projection (here, a star), that is then replicated in the 3 different positions (Middle). Right. Experimental demonstration of 50 holographic circular spots of 15 μm diameter in a 300 × 300 × 500 μm^3^ volume, adapted from Accanto et al. (2018). **(D)** GPC beam. SLM1 is used for a binary phase modulation of *Δφ* = *π*, that is then phase contrasted by the phase contrast filter (PCF), placed at the focal plane of L1. A sharp speckle-free pattern is formed at the grating plane by L2. Middle. 3 replicas for the GPC pattern in the corresponding predefined positions of the multiplexing unit. Right. Experimental demonstration of projection of 17, 12-μm diameter circular GPC spots in a 200 × 200 × 200 μm^3^ volume, adapted from Accanto et al. (2018). L3 Collimating lens.

**FIGURE 3:**
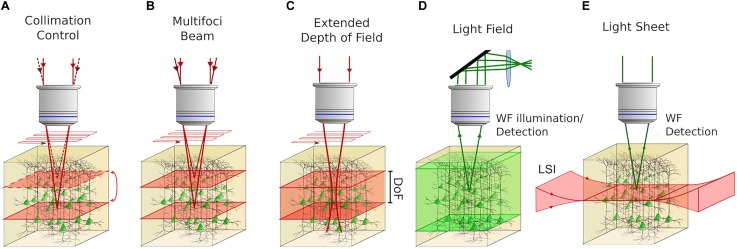
Volume imaging techniques with the possibility to be coupled with 3D photostimulation in all-optical approaches. **(A)** Control of the collimation of a scanned 2P beam at the back aperture of the objective enables sequentially addressing different focal planes in an extended volume. **(B)** Multifoci 2P techniques can be used to synchronously scan several planes of the sample providing adequate demixing strategies to distinguish the contribution coming from different planes. **(C)** Extended Depth of Field (EDF) approaches enable imaging entire volumes by 2D scan of an illumination beam, featuring an extended axial point spread function (Depth of Field; DoF). **(D)** Light Field Microscopy enables CCD-recorded volume imaging by modulating the fluorescent signal induced in the sample, upon 1P wide-field (WF) illumination by means of a system of microlenses located in the detection path and computational reconstruction algorithms. **(E)** In Light-Sheet Microscopy 2D scanless imaging is obtained by illuminating the sample with a “sheet” of light in the orthogonal direction of the microscope’s optical axis. In this way, optical sectioning is achieved and 3D volume imaging is performed by scanning the light sheet in the axial direction of the microscope. Red light indicates the excitation beam and green light indicates excited fluorescence.

Despite the flexibility of this system, an inherent limitation occurs in the maximum number of axial planes that could be addressed because of the physical tiling of the SLMs, before the quality of the holographic spots get distorted (∼6 planes) (Hernandez et al., 2016). Although this can be sufficient for a number of biological applications (Yang et al., 2016), three new studies, have recently proposed ways to increase both the number of planes and the FOE. They all used the same principle: having a beam-shaping method for creating a 2D temporally focused pattern and using a SLM at a Fourier plane of the TF system for lateral and axial multiplexing of this pattern in several positions in 3Ds via 3D-CGH (Figure 2A). 3D-CGH is used to generate arrays of 3D diffraction-limited spots, using variations of the Gerchberg-Saxton algorithm (Gerchberg and Saxton, 1972; Di Leonardo et al., 2007) to calculate the phase profile to address to the SLM.

In two of these works the light-shaping part is a TF-Gaussian beam (Pégard et al., 2017; Sun et al., 2018) (Figure 2B). In (Pégard et al., 2017) 3D scanless holographic optogenetics with temporal focusing (3D-SHOT) was used to generate a large number of temporally focused spots, each of them fitting the size of a cell soma of pyramidal neurons (i.e., ∼10–15 μm FWHM lateral size). With this approach, researchers reported the possibility to project hundreds of excitation spots in a total volume of 350 × 350 × 280 μm^3^. (Sun et al., 2018) presented a very similar approach as the one presented by (Pégard et al., 2017), using a TF experimental configuration with two gratings instead of a single grating and a lens. The beam’s lateral size was 2.5 μm in diameter and the axial FWHM of the 2P light intensity was 7.5 μm. This study did not include any biological demonstration. The authors used the generated spots in direct laser writing inside a glass, which relies on non-linear light absorption at the focus. Fabrication was conducted either on the surface or inside standard microscope glass slides. As a novelty, they showed that they were able to place focal points at high lateral proximity (4 m) with minimal interference between them. That is thanks to the pulse front tilt effect, a property inherent to TF systems where the arrival time of an ultrashort pulse in a certain plane varies across the beam profile thus creating a tilt between the pulse front and the direction perpendicular to the beam. In this way, adjacent spots can be spatially overlapped (Sun et al., 2018).

Both of these studies use Gaussian beams, which is technically simpler compared to the 3D-CGH-TF system proposed by (Hernandez et al., 2016). However, a considerable drawback of this system is the fact that the laser beam is focused on a line on the SLM used for 3D multiplexing, where the size depends on the linear dispersion of the TF system in the direction of dispersing the spectral frequencies (usually parallel to the optical table), and it equals the monochromatic beam size in the unchirped dimension (vertical to the dispersion direction). In the chirped direction, care is usually given to fill the size of the SLM liquid crystal array, while in the unchirped direction the size of the beam is a few millimeters (2–3 mm) (Durst et al., 2008; Sun et al., 2018). This imposes a restriction to the maximum laser power used before damaging the SLM (and thus to the maximum number of spots that can be projected). To overcome this limitation Pégard et al. (2017) proposed adding an extra lens before dispersing the ultrashort pulses on the TF grating, defocusing the beam on the SLM and thus increasing the illumination area. Nevertheless, the defocusing created a secondary spatial focus in the form of a line that deteriorated the axial propagation of the beam (Pégard et al., 2017; Chen et al., 2018c). In a more advanced version of this method the lens was replaced by a rotating diffuser at an image plane, after the grating. This led to an enlarged illumination of the SLM, without secondary focus effects (Mardinly et al., 2018) and Gaussian beams of 23 μm axial FWHM (axial optical Point Spread Function; PSF) (Pégard et al., 2018). 3D-SHOT was used in this case to simultaneously stimulate neurons co-expressing newly developed excitatory or inhibitory somatic opsins, ST-ChroME or IRES-ST-eGTACR1, respectively, and GCaMP6s in an all-optical configuration (about 50 neurons in three different planes extending to an axial range of 100 μm, 0.13 mW/μm^2^ or 40 mW per target for activating ST-ChroME neurons, 0.08 mW/μm^2^ or ∼6 mW per target for IRES-ST-eGTACR1 neurons, illumination with a low-repetition rate laser at 1040 nm for 1 s) (Mardinly et al., 2018).

Higher flexibility and better axial resolution was demonstrated by Accanto et al. who presented a system for multiplexed temporally focused-light shaping (MTF-light shaping), where the beam-shaping part was either 2D-CGH or GPC (Accanto et al., 2018) (Figure 2C,D). For MTF-CGH, the optical setup is the same as the one of 3D-CGH-TF (Hernandez et al., 2016), the only difference being the way the multiplexing SLM is addressed. MTF-CGH enabled investigators to generate 15-μm diameter temporally focused holographic spots on 50 independent planes on an excitation field of 300 × 300 × 500 μm^3^ and an average axial PSF of 11 μm FWHM. The theoretical FOE for the optical parameters they used was 750 × 750 × 990 μm^3^, but was experimentally limited by the size of the optics used.

In MTF-GPC the beam-shaping part was substituted by a GPC setup (Glückstad, 1996; Papagiakoumou et al., 2010) for projection of high-precision, speckle-less, temporally focused arbitrarily shaped patterns. Notably, the combination of GPC with CGH to extend GPC to 3D was previously reported (Go et al., 2011; Bañas and Glückstad, 2017) but without TF, which is essential for suppressing excitation by the out-of-focus light (Papagiakoumou et al., 2010). In MTF-GPC, characterization of 12-μm diameter TF-GPC spots showed improved axial PSF, compared to MTF-CGH (6 μm FWHM on average on a FOE of 200 × 200 × 200 μm^3^), as expected for TF-GPC patterns (Papagiakoumou et al., 2010). Similar to the Gaussian beam case, a crucial drawback remains the illumination of the multiplexing SLM with a line. To overcome this, (Accanto et al., 2018) removed the phase contrast filter and used the first SLM of their configuration to perform amplitude and phase modulation. They encoded a pattern in four different areas on the beam-shaping SLM and the pattern of each area was laterally displaced with a different prism-phase effect, such as generating four different lines on the multiplexing SLM after the beams were temporally focused. Addressing the first SLM in different areas enabled the projection of replicas of four different speckle-free patterns in a volume of 300 × 300 × 400 μm^3^, a method referred to as MTF-Multi Shapes. This strategy both increased the illumination area of the multiplexing SLM and allowed more flexibility on the shape of the projected patterns, similar to that described by (Hernandez et al., 2016).

Evidently, flexibility of the MTF-light shaping methods comes at the cost of simplicity of the optical setup and total cost. For simpler cost-effective solutions in applications where the excitation spot size and form can be predetermined, it is possible to use static lithographically fabricated phase masks (Accanto et al., 2018) to replace the first SLM in MTF-CGH, or a GPC-light shaper (Bañas et al., 2014) to replace the GPC setup in MTF-GPC.

MTF-CGH was used in a multi-cell excitation of photoactivatable GCaMP (paGCaMP) in the central nervous system of the drosophila larvae (photoconversion at 760 nm with 1.0 mW/μm^2^, illumination with trains of 10-ms pulses at 50 Hz, total illumination duration: tens of seconds up to 4 min) and to photoconvert Kaede in the zebrafish larva hindbrain (photoconversion at 800 nm with 0.4 mW/μm^2^, illumination with trains of 10-ms pulses at 50 Hz, total illumination duration 1–4 min). Parallel illumination of neurons allowed fast photoconversion in both cases, with minimal photoactivation of untargeted neighboring cells. Especially in the case of paGCaMP, neuronal processes of the targeted cells could be clearly distinguished from the background, allowing the possibility to precisely track neuronal morphology (Accanto et al., 2018).

Although the use of TF in neuronal photoactivation with parallel methods has offered the possibility to locally confine the excitation volume, such as to preserve single-cell resolution, this might not be necessary for low-scattering samples, excitation of small size cells or sparse staining. In that case, 3D-CGH alone can be used for the projection of extended light patterns in different planes (Haist et al., 1997; Hernandez et al., 2016). Thus 2P 3D-CGH spots of 6-μm diameter were used to photoactivate ChR2 in the zebrafish larval neurons, which in combination with 2P GCaMP6s Ca^2+^ imaging, enabled the identification of neuronal ensembles associated with control of tail bending (photoactivation at 920 nm, 0.2 mW/μm^2^ or 50 mW per target, circular overlapping photoactivation regions of 18 μm in diameter) (Dal Maschio et al., 2017). Moreover, the authors performed targeted photoactivatable GFP (paGFP) photoconversion to obtain a morphological reconstruction of individual functionally identified neurons (photoconversion at 750 nm with 0.25 mW/μm^2^ or 7 mW per target, 1-s illumination).

Finally, as a general comment we should note that 3D-TF methods using CGH for multiplexing the excitation spot, can produce powerful experimental configurations in volumetric FOEs, with the quality of all spots being the same across the whole FOE, since the spots multiplexed are replicas of the same single original spot. However, for volumes reaching mm-range, care should be taken to homogenize the excitation properties of the projected spots, by taking into account factors such as scattering with increasing depth, SLM-diffraction-efficiency corrections, optical aberrations due to the large defocusing of spots (objective used at its limits) or projection near the borders of the FOE, and spectral aberrations for TF occurring by cropping spectral frequencies in the optics when using large defocus (Hernandez et al., 2016).

#### Hybrid Methods

Scanning methods are alternative approaches for neuronal stimulation and have been widely used in 2P optogenetics (Rickgauer and Tank, 2009; Andrasfalvy et al., 2010; Packer et al., 2012; Prakash et al., 2012), mainly for the activation of the slow kinetics opsin, C1V1, and most of the times in a spiral trajectory (Packer et al., 2012, 2015; Carrillo-reid et al., 2016; Yang et al., 2018). They represent the simplest and most immediate solution for many laboratories, since they adopt conventional 2P scanning microscopes based on galvanometric scanners. Nevertheless, the sequential photostimulation limits the achievable temporal resolution (Papagiakoumou, 2013; Ronzitti et al., 2017b) and does not allow the simultaneous activation of multiple targets. The use of resonant scanners or acousto-optic deflectors (AODs) to increase the temporal resolution of scanning methods, is still limited by the necessary dwell time in 2P excitation, especially for slow opsins (Prakash et al., 2012). Moreover, due to their cycling at resonant frequencies (8 kHz), resonant scanners cannot provide the necessary flexibility for arbitrary excitation trajectories, like spiral scans. For simultaneous multicell activation in 3Ds, scanning microscopes can be modified to include a SLM to multiplex the beam prior to the scan via 3D-CGH (Packer et al., 2012; Yang et al., 2018). The holographic pattern, consisting of multiple near-diffraction limited spots (∼1 μm in diameter), is then scanned in spiral trajectories on an area covering the size of the cell soma (Rickgauer and Tank, 2009; Packer et al., 2015; Yang et al., 2018).

Studies using hybrid methods in all-optical configurations have so far used C1V1 as optogenetic actuator excited at 1040 nm (Packer et al., 2015; 20–80 mW per target, spirals of 20 ms, 80 MHz repetition rate laser; Yang et al., 2018; 2.2–6.0 mW per target, spirals of 20 ms, low-repetition rate amplified laser). For 3D manipulation of neurons specifically, there is currently only one study where investigators simultaneously photostimulated more than 80 neurons over 150 μm in depth in layer 2/3 of the mouse visual cortex, while simultaneously imaging the activity of the surrounding neurons with GCaMP6s (Yang et al., 2018). The authors photoactivated in three different planes in an axial range of 100 μm selected groups of somatostatin inhibitory interneurons, suppressing the response of nearby pyramidal neurons to visual stimuli in awake animals (6 mW per cell from a low-repetition rate laser, or 6 mW/μm^2^, since the surface of the illumination spot in that case is about 1 μm^2^, illumination for 2.8 s with 175 continuously repeated spirals, each lasting ∼16 ms).

#### Maximum Number of Excitation Targets

An estimate of the maximum possible number of targets to address with each approach in the framework of an all-optical experiment, presumes knowledge of the total light losses of an optical system from the laser source to the objective output, which can significantly vary from one system to another. Moreover, the power necessary per target used, can vary according to opsin type, expression level, cell health and activation depth. In general, power losses for parallel illumination methods mainly consist of losses on the SLM(s) and the diffraction grating used for TF. For hybrid methods losses are approximately 2–3 times less than those of parallel ones, since they are mainly due to the use of a single SLM. It has also been reported that parallel approaches need about twice the power used by a spiral scanned beam, to induce a neuronal response with the same properties in both cases (Picot et al., 2018; Yang et al., 2018). Thus, in principle, for the same laser source and systems carefully designed to minimize power losses, hybrid methods are supposed to outmatch parallel ones about 4 times in the maximum number of cells possibly activated (without considering photodamage limits). From what is reported so far in literature, the maximum number of cells that have been simultaneously activated with parallel approaches is 50 neurons (Mardinly et al., 2018), while for hybrid methods this number is reported to be up to 80 (Yang et al., 2018). In the first case, the authors clearly state that they were limited by the available power of their laser system. However, current advances in laser technology can provide fiber amplified systems that deliver up to 60 W maximum average power, allowing for the possibility to greatly increase the above reported numbers.

A fundamental difference between parallel and hybrid 3D multi-target photoactivation methods is the eventual photodamage effects that one can induce by increasing the number of targets and thus the amount of light that is sent to the tissue. As presented in the previously reported cases, in general, parallel illumination approaches use lower illumination intensity [<0.4 mW/μm^2^ independently on the opsin type, see also (Chen et al., 2018b)] but higher total average power per target (e.g., for 2P *in vivo* activation 10–45 mW when amplified laser pulses of tens of μJ pulse energy are used from low-repetition rate fiber amplifiers, 30–90 mW when nJ-energy pulses are used from MHz-repetition rate oscillators) than scanning approaches (Chen et al., 2018b; Forli et al., 2018; Mardinly et al., 2018), making them more vulnerable to heating, i.e., linear photodamage. On the other hand, scanning (or hybrid) methods use high intensity (2–6 mW/μm^2^) focused beams of low total average power (Yang et al., 2018) (although average powers in the range of 20–80 mW were reported (Packer et al., 2015) when nJ-energy pulses were used from MHz-repetition rate oscillators), making them vulnerable to non-linear photodamage.

For non-linear photodamage, the damage threshold was shown to be inversely proportional to the pulse duration and proportional to the square of the mean power (König et al., 1999). It has been evaluated on the basis of morphological damage for CHO (Chinese Hamster Ovarian) cells to 0.1 J/cm^2^ (König et al., 1999), or tissue ablation for porcine cornea (Olivié et al., 2008) to 1.5–2.2 J/cm^2^ for 800–1000 nm. For comparison, an intensity of 80 mW/μm^2^ at 80 MHz pulse repetition rate corresponds to a fluence of 0.1 J/cm^2^. No relevant studies exist for the mouse brain. A recent study on tissue heating, took the standard illumination parameters for either parallel or scanning methods into consideration, showing that the local temperature rise on a target area did not exceed the physiological limits in both cases (<1 K) (Picot et al., 2018). Specifically, to generate an action potential *in vivo*, with a holographic spot of 12-μm diameter at a depth of ∼150 μm, illuminating a neuron for 3 ms, at 1030 nm and 0.1 mW/μm^2^, the average temperature rise over the spot’s surface, is estimated to 0.3 K. Furthermore, comparing the temperature rise for experimental conditions able to generate action potentials with latency in the range of 2–10 ms *in vitro*, it was found that for a holographic spot (3-ms illumination, 0.2 mW/μm^2^) the average rise was 1 K, while for a focused beam in a spiral trajectory (3-ms illumination, 31 mW/μm^2^) the mean temperature rise was <0.5 K, and the local rise at the center of the spiral was again ∼1 K (Picot et al., 2018). For multi-target excitation, what remains critical is the distance between the different targets: for spots placed at an average distance from their nearest neighbor greater than the thermal diffusion length in tissue $l_{\text{th}}=\sqrt{6Dt}$, where *D* is the thermal diffusion constant [140 μm^2^/ms (Yizhar et al., 2011), and *t* the evolved time], the temperature rise remains comparable to the case of the isolated spot (for 3-ms illumination duration, holographic stimulation at an intensity ≤0.2 mW/μm^2^ enables keeping the induced temperature rise <2 K for activating 100 cells whose inter-soma distance was larger than the thermal diffusion length, which in that case was ∼50 μm). Otherwise, the heat load starts to significantly increase locally (Picot et al., 2018). Moreover, in terms of illumination duration, prolonged stimulation (>1 min) was found to induce substantial brain heating (6–8°C) (Mardinly et al., 2018).

Notably, the considerations of the study by Picot et al. (2018) indicate that the optimal laser repetition rate for 2P optogenetics depends on the adopted illumination method: the very low excitation intensity used in parallel illumination, allows to neglect non-linear damage effects and privileges using low (500 kHz–2 MHz) repetition rate lasers to minimize heating through linear absorption. Scanning approaches on the other hand, require high excitation intensity but enable more efficient heat dissipation because of their small beam spot size; therefore, for short illumination times, a higher repetition rate laser (Ji et al., 2008) should be preferred in order to minimize peak-power-sensitive damages.

### 3D Imaging

In order to combine photostimulation and functional imaging over large neuronal populations in extended volumes, it is necessary to elaborate strategies to decouple and independently control the photostimulation and the imaging plane. An exhaustive presentation of the existing imaging techniques have recently been presented in literature (Ji et al., 2016; Yang and Yuste, 2017). Here we will discuss the techniques that can be combined with 3D photoactivation, in volumetric all-optical studies.

Adoption of approaches for 3D imaging, involving fast mechanical axial movements of the objective lens with piezoelectric positioners (Grewe et al., 2010; Katona et al., 2011), in combination with 3D photoactivation methods can be rather challenging for an independent control of the stimulation and imaging planes. In that case, the microscope objective is shared by both photostimulation and imaging paths, and a 3D all-optical configuration would require a simultaneous readjustment of the axial position of the photostimulation foci, to compensate for the objective shifts. Since 3D photoactivation methods use a SLM to project the excitation spots/patterns to different axial planes at a maximum refresh rate of 3 ms (Yang et al., 2016), a combination of fast piezo-repositioning approaches is feasible in cases where imaging is done in few discrete axial planes (Cossell et al., 2015; Peron et al., 2015; Seelig and Jayaraman, 2015), but not possible when objectives need to be moved over an extended volume in a quasi-continuous way (Grewe et al., 2010; Katona et al., 2011).

Strategies involving the fast repositioning of the imaging focus by modulating the imaging beam divergence (Figure 3A), appear to be more convenient for all-optical volume investigations. This can be done by introducing a *lens-effect* in an upstream location of the imaging path, possibly in a plane conjugated to the objective back aperture to obtain a telecentric system. Clearly, the control of laser divergence must be fast enough to be compatible with functional imaging rates. Few technologies are commercially available for high-speed focus control through lenses. They are based either on the curvature change of a flexible-membrane electrically controlled lens (usually referred as Electrically Tunable Lens; ETL) (Grewe et al., 2011) or on ultrasounds propagating in a confined fluid, resulting in a tunable index of refraction gradient lens [usually referred as Tunable Acoustic Gradient (TAG) lens] that behaves like an aspheric lens (Mermillod-Blondin et al., 2008). ETLs have been mainly driven in stepping mode, enabling a ≈15 ms refocus time and can be electronically synchronized with the two-photon scanning imaging acquisition (Grewe et al., 2011). They have been successfully applied in 3D all-optical experiments, enabling simultaneous two-photon imaging and photostimulation on three planes axially spanning over 100 μm in mammals (Yang et al., 2018) and on five planes axially spanning over 32 μm in zebrafish (Dal Maschio et al., 2017). In TAG varifocal lenses, the optical power varies continuously at resonant frequencies, thus enabling much higher speeds with a ≈1 μs switching time, but they require careful control of oscillations through optical-phase locking (Kong et al., 2015). Volume imaging is built up by stacks of *xz* planes (where the fast-axis is along the axial direction) resulting in high-rate volume imaging ranging between 14 Hz (375 × 112 × 130 μm^3^) and 56 Hz (60 × 4 × 30 μm^3^).

Alternatively, SLMs or AODs can be used to dynamically control the degree of divergence of the imaging beam. SLM permits wavefront modulation resulting in fast beam refocusing (Dal Maschio et al., 2011) with refreshing rates up to 300 Hz. Importantly, in this case modulation is not limited to beam refocusing but can potentially be combined with more complex wavefront modulations, to correct optical aberrations (Booth, 2014) or to optimize the signal to noise ratio by targeting illumination of the cell (in this latter case in a CCD detection scheme) (Foust et al., 2015; Bovetti et al., 2017; Tanese et al., 2017). In the latter 2P near diffraction-limited stationary laser spots generated through CGH were used to perform scanless high-speed imaging of GCaMP6 activity in neurons *in vivo* on a CCD camera (Bovetti et al., 2017), or voltage imaging on dendritic spines (Tanese et al., 2017). CGH-shaped excitation was also used to improve the signal to noise ratio in voltage imaging experiments of dendrites and axons (Foust et al., 2015).

AODs can also be used for fast 3D beam repositioning. Using two pairs of orthogonal AODs addressed with counter-propagating acoustic waves of linearly varying frequency (chirped waves), it is possible to impress a precise *xy* radial deflection (determined by the center frequencies of the waves) and *z* axial displacement (determined by the amount of chirp) to the illumination beam. Being completely inertia-free, AOD systems can achieve very short commutation (24.5 μs for 3D random access) and dwell (0.05–1.0 μs) times (Kirkby et al., 2010; Nadella et al., 2016). This makes AODs especially well-suited for high-rate random access point- or line-scanning imaging. For instance, in (Katona et al., 2012) the authors recorded responses from a population of individual neurons and glial cells, in the visual cortex of adult anesthetized mice, by automated tissue-drift compensation performed plane by plane, when obtaining a reference z-stack or between 3D random-access scans. They recorded spontaneous activity within 400 × 400 × 500 μm^3^ at a frequency up to 56 Hz. In another example (Nadella et al., 2016), researchers performed random-access patch imaging of neurons in layer 2/3 of primary visual cortex in an awake behaving mouse at 50 Hz, as well as the simultaneous dendritic and somatic imaging of pyramidal neurons in the visual cortex of awake mice at 27.9 Hz, by applying *post hoc* movement correction of images. The downside of such systems is that the combination of four AODs in a series is associated with power losses up to 75% (Reddy et al., 2008; Nadella et al., 2016) and requires strategies to compensate the temporal and spatial dispersion (Katona et al., 2012). However, recent developments in the AOD technology allow more efficient and uniform light transmission over larger scan angles when focusing away from the nominal focal plane of the objective (Nadella et al., 2016).

Remote focusing is another approach enabling fast sequential imaging of axially separated planes. It allows remote axial shifting the imaging beam by integrating a classical raster scanning system with an axial scan unit, which comprises of an objective lens and a lightweight mirror (Botcherby et al., 2007, 2012). Since the mirror is imaged on the sample plane, its oscillations are translated in a rapid change of focus in the sample, without physically moving the imaging objective lens. Fast oscillations are enabled by using a custom-built actuator constructed with a pair of galvanometer motors to scan the mirror in the kHz range. Importantly, as the two objectives are disposed to modify the beam wavefront with equal and opposite aberrations, the microscope is resilient to the systematic aberrations introduced by diverging beams that yield large focus shifts. Remote focusing enabled volume imaging of extracellular electrically induced calcium transients in OGB-loaded neurons up to 1 kHz, over a depth of 60 μm (Botcherby et al., 2012). Of note, other systems using remote focusing units, have used voice coil motors to drive the movable mirror at high speed (Rupprecht et al., 2016; Sofroniew et al., 2016).

Other volume imaging approaches, potentially compatible with 3D photostimulation, are based on multi-foci beams sent in parallel on the sample, imaging different planes simultaneously (Figure 3B), provided that *ad hoc* read-out demultiplexing strategies are adopted, to distinguish the signal coming from those planes. One possible strategy involves temporally multiplexed beams. Pulsed beams can be sent at different foci with different time delays. If the delays are superior to the fluorescence lifetime decay (in the range of few ns) and inferior to the inter-pulse interval, the fluorescence signal originating from different locations, can be distinguished by temporally demultiplexing the detected signal (Amir et al., 2007; Cheng et al., 2011). Other strategies rely on computational demultiplexing algorithms, which permit simultaneous multi-foci imaging, without introducing any temporal shifts among the beams. In this case, a priori knowledge of the cells distribution and sparsity of cortical neuronal activity, allow demixing signals from different planes, using independent component analysis or non-negative matrix factorization algorithms (Yang et al., 2016).

Further strategies use extended depth of field (EDF) imaging, where the illumination PSF elongates axially (Botcherby et al., 2006). Since several layers of cells are encompassed within the PSF (Figure 3C), a lateral scan of such a beam is equivalent to projecting a stack of axially displaced layers in a single plane. Volume imaging is thus enabled at speeds equal to scan-based planar imaging. Very high-volume rates of functional imaging can thus be obtained, provided that neural activity comes from the sparse distribution of neurons, that do not significantly overlap axially and an *a priori* high-resolution mapping of the cells position in the volume, is acquired. Bessel beam based EDF functional volume imaging has been reported *in vivo* at 30 Hz for volumes extending up to 160 μm (Lu et al., 2017).

EDF imaging can also be obtained by engineering the detection PSF. Here, the strategy is conceptually reversed compared to previous approaches: instead of attaining volume imaging by modifying the illumination beam, axial discrimination is achieved by modulating the detected fluorescent signal. Similar to the excitation PSF, the detection PSF can be phase-only modulated with a transparent static phase mask placed at a Fourier plane of the detection path, so that it does not disturb the numerical aperture and the photon throughput of the system (no photon losses). Imaging can then be performed with a CCD and computational tools can be used to recover image information over the entire extended depth of field, as in the case of the elongated excitation PSF. Researchers have shown that the use of cubic phase masks in such configurations, in combination with CGH-based target illumination, allows for simultaneous imaging of fluorescence signals arising from different 3D targeted points (Quirin et al., 2013). Moreover, an *a priori* information of the origin of the fluorescence signal, through targeted excitation with CGH spots, can remove any ambiguity arising from imaging unknown objects with extended axial features (Quirin et al., 2013). Here, the strategy is conceptually reversed compared to previous approaches: instead of attaining volume imaging by modifying the illumination beam, axial discrimination is achieved by modulating the detected fluorescent signal.

Alternatively, volumetric imaging can be obtained in a wide-field illumination configuration using Light Field Microscopy (LFM) (Broxton et al., 2013) (Figure 3D). A series of micro lenses are placed at the native image plane (i.e., the plane where a camera is put in standard wide-field configurations) and a relay lens system is used to reimage the lenslets’ back focal plane onto a camera (Broxton et al., 2013). Since in-focus and out-of-focus light results in different patterns at the camera, axial localization of the emitters in a sample volume can be obtained by computationally processing the image. The volumetric imaging speed is only limited by the CCD acquisition rate. Despite the high temporal performances, the application of LFM has been restricted to semi-transparent tissues due to scattering limitations (Broxton et al., 2013; Cohen et al., 2014; Prevedel et al., 2014). However, it has recently been proposed in a computational imaging approach, where it is integrated with high-dimensional, structured statistics enabling fast volumetric acquisition *in vivo* in the brains of mammals (Grosenick et al., 2017). If coupled with 3D photoactivation, particular attention needs to be paid when considering photoactivation cross-talk, as contamination induced by a single-photon wide-field imaging beam, may not be negligible.

Finally, light-sheet microscopy (LSM) represents another volumetric imaging approach (Figure 3E) particularly suited to whole-brain imaging of small-scale organisms (Keller and Ahrens, 2015; Power and Huisken, 2017), at single-cell imaging resolution (Ahrens et al., 2013). In this case, optical sectioning of the specimen is obtained in a conventional wide-field detection scheme, with an orthogonal illumination of the sample by means of a thin “sheet” of light coming from the side. Since excitation only yields in an axially confined planar portion of the sample, optically sectioned video-rate imaging of specific planes is enabled simply by using a common camera-based detection. 3D photostimulation coupling could be, e.g., envisaged by delivering photostimulation light through the high-NA detection objective. All-optical 3D functional investigations might then be obtained by adopting those LSM volumetric strategies involving axial light-sheet repositioning and varifocal ETL-based detection (Fahrbach et al., 2013) or cubic phase-based extended depth of field detection approaches, combined with imaging deconvolution (Olarte et al., 2015; Quirin et al., 2016). It is worth mentioning that, since the side-on light-sheet needs to uniformly excite a large portion of tissue, LSM is chiefly adopted for imaging of low-scattering media, even if imaging in relatively opaque tissues have been demonstrated in double-sided illumination and detection arrangements (Tomer et al., 2012; Lemon et al., 2015; Ezpeleta et al., 2016). Interestingly, double-sided illumination can be potentially used with one low-NA objective to generate the light-sheet and the other opposite-sided objective chosen with a high NA to address the 3D photostimulation patterns. At last, it should also be considered, that the orthogonal disposition of illumination and detection objectives, ultimately limit the geometric accessibility to the sample compared to other techniques relying on a single objective.

## Outlook

From this overview of the methods for 3D photoactivation and imaging, it is evident that these domains have tremendously advanced over the last few years. However, the combination of all-optical approaches is so far limited to use 2P scanning imaging modalities with an ETL (Dal Maschio et al., 2017; Mardinly et al., 2018; Yang et al., 2018), which is the most straightforward method for multiplane imaging, from those presented.

Nevertheless, the first steps have now been completed and we are entering an era where there will be an increasing demand for high-performance all-optical methods to tackle more complex biological questions. This will certainly prompt further developments for all-optical strategies in large excitation volumes and multi-area microscopes, where it will be possible, for instance, to activate a population of neurons in one area while monitoring the effects in another area of the brain (Lecoq et al., 2014; Chen et al., 2016). Furthermore, for imaging and photoactivation in large depths, development of micro-endoscopes based on miniaturized optics (Zong et al., 2017; Zou et al., 2017; Ozbay et al., 2018), able to perform all-optical manipulations, and use of three-photon excitation (Horton et al., 2013; Chen et al., 2018a; Rodríguez et al., 2018), can be envisioned.

## Author Contributions

EP conceived and organized the structure of the manuscript. EP and ER wrote the manuscript and prepared the figures. VE revised and contributed in writing the manuscript.

## Conflict of Interest Statement

The authors declare that the research was conducted in the absence of any commercial or financial relationships that could be construed as a potential conflict of interest. The handling Editor declared a past co-authorship with the authors.

## References

[B1] Accanto N. Molinier C. Tanese D. Ronzitti E. Newman Z. L. Wyart C. (2018). Multiplexed temporally focused light shaping for high-resolution multi-cell targeting. *Optica* 5 1478–1491. 10.1364/OPTICA.5.001478

[B2] Ahrens M. B. Orger M. B. Robson D. N. Li J. M. Keller P. J. (2013). Whole-brain functional imaging at cellular resolution using light-sheet microscopy. *Nat. Methods* 10 413–420. 10.1038/nmeth.2434 23524393

[B3] Airan R. D. Thompson K. R. Fenno L. E. Bernstein H. Deisseroth K. (2009). Temporally precise in vivo control of intracellular signalling. *Nature* 458 1025–1029. 10.1038/nature07926 19295515

[B4] Amir W. Carriles R. Hoover E. E. Durfee C. G. Squier J. A. (2007). Simultaneous imaging of multiple focal planes in scanning two-photon absorption microscope. *Opt. Lett.* 32 1731–1733. 10.1117/12.731868 17572762

[B5] Andrasfalvy B. K. Zemelman B. V. Tang J. Vaziri A. (2010). Two-photon single-cell optogenetic control of neuronal activity by sculpted light. *Proc. Natl. Acad. Sci. U.S.A.* 107 11981–11986. 10.1073/pnas.1006620107 20543137 PMC2900666

[B6] Anselmi F. Ventalon C. Begue A. Ogden D. Emiliani V. Bègue A. (2011). Three-dimensional imaging and photostimulation by remote-focusing and holographic light patterning. *Proc. Natl. Acad. Sci. U.S.A.* 108 19504–19509. 10.1073/pnas.1109111108 22074779 PMC3241782

[B7] Bañas A. Glückstad J. (2017). Holo-GPC: holographic generalized phase contrast. *Opt. Commun.* 392 190–195. 10.1016/j.optcom.2017.01.036

[B8] Bañas A. Palima D. Villangca M. Aabo T. Glückstad J. (2014). GPC light shaper for speckle-free one- and two- photon contiguous pattern excitation. *Opt. Express* 7102 5299–5310. 10.1364/OE.22.005299 24663871

[B9] Becker Y. Unger E. Fichte M. A. H. Gacek D. A. Dreuw A. Wachtveitl J. (2018). A red-shifted two-photon-only caging group for three-dimensional photorelease. *Chem. Sci.* 9 2797–2802. 10.1039/C7SC05182D 29732066 PMC5914290

[B10] Bègue A. Papagiakoumou E. Leshem B. Conti R. Enke L. Oron D. (2013). Two-photon excitation in scattering media by spatiotemporally shaped beams and their application in optogenetic stimulation. *Biomed. Opt. Express* 4 2869–2879. 10.1364/BOE.4.002869 24409387 PMC3862165

[B11] Booth M. J. (2014). Adaptive optical microscopy: the ongoing quest for a perfect image. *Light Sci. Appl.* 3:e165 10.1038/lsa.2014.46

[B12] Botcherby E. J. Juskaitis R. Booth M. J. Wilson T. (2007). Aberration-free optical refocusing in high numerical aperture microscopy. *Opt. Lett.* 32 2007–2009. 10.1364/OL.32.002007 17632625

[B13] Botcherby E. J. Smith C. W. Kohl M. M. Débarre D. Booth M. J. Juskaitis R. (2012). Aberration-free three-dimensional multiphoton imaging of neuronal activity at kHz rates. *Proc. Natl. Acad. Sci. U.S.A.* 109 2919–2924. 10.1073/pnas.1111662109 22315405 PMC3286923

[B14] Botcherby E. J. J. Juškaitis R. Wilson T. Botcherby E. J. J. Jus R. Juskaitis R. (2006). Scanning two photon fluorescence microscopy with extended depth of field. *Opt. Commun.* 268 253–260. 10.1016/j.optcom.2006.07.026 23609714

[B15] Bovetti S. Moretti C. Zucca S. Dal Maschio M. Bonifazi P. Fellin T. (2017). Simultaneous high-speed imaging and optogenetic inhibition in the intact mouse brain. *Sci. Rep.* 7:40041. 10.1038/srep40041 28053310 PMC5215385

[B16] Broxton M. Grosenick L. Yang S. Cohen N. Andalman A. Deisseroth K. (2013). Wave optics theory and 3-D deconvolution for the light field microscope. *Opt. Express* 21 25418–25439. 10.1364/OE.21.025418 24150383 PMC3867103

[B17] Carrillo-reid L. Yang W. Bando Y. Peterka D. S. Yuste R. (2016). Imprinting and recalling cortical ensembles. *Science* 353 691–694. 10.1126/science.aaf7560 27516599 PMC5482530

[B18] Carroll E. C. Berlin S. Levitz J. Kienzler M. A. Yuan Z. Madsen D. (2015). Two-photon brightness of azobenzene photoswitches designed for glutamate receptor optogenetics. *Proc. Natl. Acad. Sci. U.S.A.* 112 E776–E785. 10.1073/pnas.1416942112 25653339 PMC4343171

[B19] Chaigneau E. Ronzitti E. Gajowa A. M. Soler-Llavina J. G. Tanese D. Brureau Y. B. A. (2016). Two-photon holographic stimulation of ReaChR. *Front. Cell. Neurosci.* 10:234. 10.3389/fncel.2016.00234 27803649 PMC5067533

[B20] Chen B. Huang X. Gou D. Zeng J. Chen G. Pang M. (2018a). Rapid volumetric imaging with Bessel-Beam three-photon microscopy. *Biomed. Opt. Express* 9 1992–2000. 10.1364/BOE.9.001992 29675334 PMC5905939

[B21] Chen I. W. Papagiakoumou E. Emiliani V. (2018b). Towards circuit optogenetics. *Curr. Opin. Neurobiol.* 50 179–189. 10.1016/j.conb.2018.03.008 29635216 PMC6027648

[B22] Chen I.-W. Ronzitti E. Lee R. B. Daigle L. T. Zeng H. Papagiakoumou E. (2018c). Parallel holographic illumination enables sub-millisecond two-photon optogenetic activation in mouse visual cortex in vivo. *bioRxiv* [Preprint]. 10.1101/250795

[B23] Chen J. L. Voigt F. F. Javadzadeh M. Krueppel R. Helmchen F. (2016). Long-Range population dynamics of anatomically defined neocortical networks. *eLife* 5:e14679. 10.7554/eLife.14679 27218452 PMC4929001

[B24] Cheng A. Gonçalves J. T. Golshani P. Arisaka K. Portera-Cailliau C. (2011). Simultaneous two-photon calcium imaging at different depths with spatiotemporal multiplexing. *Nat. Methods* 8 139–142. 10.1038/nmeth.1552 21217749 PMC3076599

[B25] Cohen N. Yang S. Andalman A. Broxton M. Grosenick L. Deisseroth K. (2014). Enhancing the performance of the light field microscope using wavefront coding. *Opt. Express* 22 24817–24839. 10.1364/OE.22.024817 25322056 PMC4247191

[B26] Cossell L. Iacaruso M. F. Muir D. R. Houlton R. Sader E. N. Ko H. (2015). Functional organization of excitatory synaptic strength in primary visual cortex. *Nature* 518 399–403. 10.1038/nature14182 25652823 PMC4843963

[B27] Dal Maschio M. De Stasi A. M. Benfenati F. Fellin T. (2011). Three-dimensional in vivo scanning microscopy with inertia-free focus control. *Opt. Lett.* 36 3503–3505. 10.1364/OL.36.003503 21886258

[B28] Dal Maschio M. Difato F. Beltramo R. Blau A. Benfenati F. Fellin T. (2010). Simultaneous two-photon imaging and photo-stimulation with structured light illumination. *Opt. Express* 18 18720–18731. 10.1364/OE.18.018720 20940765

[B29] Dal Maschio M. Donovan J. C. Helmbrecht T. O. Baier H. (2017). Linking neurons to network function and behavior by two-photon holographic optogenetics and volumetric imaging. *Neuron* 94 774–789.e5. 10.1016/j.neuron.2017.04.034 28521132

[B30] Dana H. Shoham S. (2012). Remotely scanned multiphoton temporal focusing by axial grism scanning. *Opt. Lett.* 37 2913–2915. 10.1364/OL.37.002913 22825176

[B31] Di Leonardo R. Ianni F. Ruocco G. (2007). Computer generation of optimal holograms for optical trap arrays. *Opt. Express* 15 1913–1922. 10.1364/OE.15.001913 19532430

[B32] Ducros M. Goulam Houssen Y. Bradley J. de Sars V. Charpak S. (2013). Encoded multisite two-photon microscopy. *Proc. Natl. Acad. Sci. U.S.A.* 110 13138–13143. 10.1073/pnas.1307818110 23798397 PMC3740851

[B33] Durst M. E. Zhu G. Xu C. (2006). Simultaneous spatial and temporal focusing for axial scanning. *Opt. Express* 14 12243–12254. 10.1364/OE.14.012243 19529653

[B34] Durst M. E. Zhu G. Xu C. (2008). Simultaneous spatial and temporal focusing in nonlinear microscopy. *Opt. Commun.* 281 1796–1805. 10.1016/j.optcom.2007.05.071 18496597 PMC2390847

[B35] Emiliani V. Cohen A. E. Deisseroth K. Häusser M. (2015). All-optical interrogation of neural circuits. *J. Neurosci.* 35 13917–13926. 10.1523/JNEUROSCI.2916-15.2015 26468193 PMC4604230

[B36] Ezpeleta E. Zurutuza U. Hidalgo J. M. G. (2016). Using personality recognition techniques to improve Bayesian spam filtering. *Proces. Leng. Nat.* 57 125–132. 10.1038/nmeth.2064 22660739

[B37] Fahrbach F. Voigt F. Schmid B. Helmchen F. Huisken J. (2013). Rapid 3D light-sheet microscopy with a tunable lens. *Opt. Express* 21 1963–1975. 10.1364/OE.21.021010 24103973

[B38] Forli A. Vecchia D. Binini N. Succol F. Bovetti S. Moretti C. (2018). Two-photon bidirectional control and imaging of neuronal excitability with high spatial resolution in vivo. *Cell Rep.* 22 2809–2817. 10.1016/j.celrep.2018.02.063 29539433 PMC5863087

[B39] Förster D. Dal Maschio M. Laurell E. Baier H. (2017). An optogenetic toolbox for unbiased discovery of functionally connected cells in neural circuits. *Nat. Commun.* 8:116. 10.1038/s41467-017-00160-z 28740141 PMC5524645

[B40] Foust A. J. Zampini V. Tanese D. Papagiakoumou E. Emiliani V. (2015). Computer-generated holography enhances voltage dye fluorescence discrimination in adjacent neuronal structures. *Neurophotonics* 2:021007. 10.1117/1.NPh.2.2.021007 26157998 PMC4478842

[B41] Gerchberg R. W. Saxton W. O. (1972). A practical algorithm for the determination of the phase from image and diffraction pictures. *Optik* 35 237–246.

[B42] Glückstad J. (1996). Phase contrast image synthesis. *Opt. Commun.* 130 225–230. 10.1016/0030-4018(96)00339-2

[B43] Go M. A. Ng P.-F. Bachor H. A. Daria V. R. (2011). Optimal complex field holographic projection. *Opt. Lett.* 36 3073–3075. 10.1364/OL.36.003073 21847164

[B44] Go M. A. Stricker C. Redman S. Bachor H.-A. A. Daria V. R. (2012). Simultaneous multi-site two-photon photostimulation in three dimensions. *J. Biophotonics* 5 745–753. 10.1002/jbio.201100101 22345073

[B45] Göbel W. Kampa B. M. Helmchen F. Go W. (2007). Imaging cellular network dynamics in three dimensions using fast 3D laser scanning. *Nat. Methods* 4 73–79. 10.1038/NMETH989 17143280

[B46] Grewe B. F. Langer D. Kasper H. Kampa B. M. Helmchen F. (2010). High-speed in vivo calcium imaging reveals neuronal network activity with near-millisecond precision. *Nat. Methods* 7 399–405. 10.1038/nmeth.1453 20400966

[B47] Grewe B. F. Voigt F. F. van ’t Hoff M. Helmchen F. van ’t Hoff M. Helmchen F. (2011). Fast two-layer two-photon imaging of neuronal cell populations using an electrically tunable lens. *Biomed. Opt. Express* 2 2035–2046. 10.1364/BOE.2.002035 21750778 PMC3130587

[B48] Grosenick L. M. Broxton M. Kim C. K. Liston C. Poole B. Yang S. (2017). Identification of cellular-activity dynamics across large tissue volumes in the mammalian brain. *bioRxiv* [Preprint]. 10.1101/132688

[B49] Guruge C. Ouedraogo Y. P. Comitz R. L. Ma J. Losonczy A. Nesnas N. (2018). Improved synthesis of caged glutamate and caging each functional group. *ACS Chem. Neurosci.* 9 2713–2727. 10.1021/acschemneuro.8b00152 29750497 PMC6294478

[B50] Haist T. Schönleber M. Tiziani H. (1997). Computer-generated holograms from 3D-objects written on twisted-nematic liquid crystal displays. *Opt. Commun.* 140 299–308. 10.1016/S0030-4018(97)00192-2

[B51] Herlitze S. Landmesser L. T. (2007). New optical tools for controlling neuronal activity. *Curr. Opin. Neurobiol.* 17 87–94. 10.1016/j.conb.2006.12.002 17174547

[B52] Hernandez O. Papagiakoumou E. Tanese D. Fidelin K. Wyart C. Emiliani V. (2016). Three-dimensional spatiotemporal focusing of holographic patterns. *Nat. Commun.* 7:11928. 10.1038/ncomms11928 27306044 PMC4912686

[B53] Horton N. G. Wang K. Kobat D. Clark C. G. Wise F. W. Schaffer C. B. (2013). In vivo three-photon microscopy of subcortical structures within an intact mouse brain. *Nat. Photonics* 7 205–209. 10.1038/nphoton.2012.336 24353743 PMC3864872

[B54] Isobe K. Hashimoto H. Suda A. Kannari F. Kawano H. Mizuno H. (2010). Measurement of two-photon excitation spectrum used to photoconvert a fluorescent protein (Kaede) by nonlinear Fourier-transform spectroscopy. *Biomed. Opt. Express* 1 687–693. 10.1364/BOE.1.000687 21258500 PMC3017996

[B55] Ji N. Freeman J. Smith S. L. (2016). Technologies for imaging neural activity in large volumes. *Nat. Neurosci.* 19 1154–1164. 10.1038/nn.4358 27571194 PMC5244827

[B56] Ji N. Magee J. C. Betzig E. (2008). High-speed, low-photodamage nonlinear imaging using passive pulse splitters. *Nat. Methods* 5 197–202. 10.1038/nmeth.1175 18204458

[B57] Katona G. Kaszás A. Turi G. F. Hájos N. Tamás G. Vizi E. S. (2011). Roller Coaster Scanning reveals spontaneous triggering of dendritic spikes in CA1 interneurons. *Proc. Natl. Acad. Sci. U.S.A.* 108 2148–2153. 10.1073/pnas.1009270108 21224413 PMC3033272

[B58] Katona G. Szalay G. Maák P. Kaszás A. Veress M. Hillier D. (2012). Fast two-photon in vivo imaging with three-dimensional random-access scanning in large tissue volumes. *Nat. Methods* 9 201–208. 10.1038/nMeth.1851 22231641

[B59] Keller P. J. Ahrens M. B. (2015). Visualizing whole-brain activity and development at the single-cell level using light-sheet microscopy. *Neuron* 85 462–483. 10.1016/j.neuron.2014.12.039 25654253

[B60] Kim E. H. Chin G. Rong G. Poskanzer K. E. Clark H. A. (2018). Optical probes for neurobiological sensing and imaging. *Acc. Chem. Res.* 51 1023–1032. 10.1021/acs.accounts.7b00564 29652127 PMC6128814

[B61] Kirkby P. A. Srinivas Nadella K. M. Silver R. A. (2010). A compact Acousto-optic lens for 2D and 3D femtosecond based 2-photon microscopy. *Opt. Express* 18 13721–13745. 10.1364/OE.18.013720 20588506 PMC2948528

[B62] Klapoetke N. C. Murata Y. Kim S. S. Pulver S. R. Birdsey-Benson A. Cho Y. K. (2014). Independent optical excitation of distinct neural populations. *Nat. Methods* 11 338–346. 10.1038/nmeth.2836 24509633 PMC3943671

[B63] Kong L. Tang J. Little J. P. Yu Y. Lämmermann T. Lin C. P. (2015). Continuous volumetric imaging via an optical phase-locked ultrasound lens. *Nat. Methods* 12 759–762. 10.1038/nmeth.3476 26167641 PMC4551496

[B64] König K. Becker T. W. Fischer P. Riemann I. Halbhuber K.-J. (1999). Pulse-length dependence of cellular response to intense near-infrared laser pulses in multiphoton microscopes. *Opt. Lett.* 24 113–115. 10.1364/OL.24.000113 18071425

[B65] Kwon T. Sakamoto M. Peterka D. S. Yuste R. (2017). Attenuation of synaptic potentials in dendritic spines. *Cell Rep.* 20 1100–1110. 10.1016/j.celrep.2017.07.012 28768195 PMC5709047

[B66] Lecoq J. Savall J. Vuèiniæ D. Grewe B. F. Kim H. Li J. Z. (2014). Visualizing mammalian brain area interactions by dual-axis two-photon calcium imaging. *Nat. Neurosci.* 17 1825–1829. 10.1038/nn.3867 25402858 PMC5289313

[B67] Lemon W. C. Pulver S. R. Höckendorf B. McDole K. Branson K. Freeman J. (2015). Whole-central nervous system functional imaging in larval *Drosophila*. *Nat. Commun.* 6:7924. 10.1038/ncomms8924 26263051 PMC4918770

[B68] Leshem B. Hernandez O. Papagiakoumou E. Emiliani V. Oron D. (2014). When can temporally focused excitation be axially shifted by dispersion? *Opt. Express* 22 7087–7098. 10.1364/OE.22.007087 24664057

[B69] Levitz J. Pantoja C. Gaub B. Janovjak H. Reiner A. Hoagland A. (2013). Optical control of metabotropic glutamate receptors. *Nat. Neurosci.* 16 507–516. 10.1038/nn.3346 23455609 PMC3681425

[B70] Liesener J. Reicherter M. Haist T. Tiziani H. J. (2000). Multi-functional optical tweezers using computer-generated holograms. *Opt. Commun.* 185 77–82. 10.1016/S0030-4018(00)00990-1

[B71] Lim D. Chu K. K. Mertz J. (2008). Wide-field fluorescence sectioning with hybrid speckle and uniform-illumination microscopy. *Opt. Lett.* 33 1819–1821. 10.1364/OL.33.001819 18709098

[B72] Lu R. Sun W. Liang Y. Kerlin A. Bierfeld J. Seelig J. D. (2017). Video-rate volumetric functional imaging of the brain at synaptic resolution. *Nat. Neurosci.* 20 620–628. 10.1038/nn.4516 28250408 PMC5374000

[B73] Lutz C. Otis T. S. DeSars V. Charpak S. Digregorio D. A. Emiliani V. (2008). Holographic photolysis of caged neurotransmitters. *Nat. Methods* 5 821–827. 10.1038/nmeth.1241 19160517 PMC2711023

[B74] Mardinly A. R. Oldenburg I. A. Pégard N. C. Sridharan S. Lyall E. H. Chesnov K. (2018). Precise multimodal optical control of neural ensemble activity. *Nat. Neurosci.* 21 881–893. 10.1038/s41593-018-0139-8 29713079 PMC5970968

[B75] Mermillod-Blondin A. McLeod E. Arnold C. B. (2008). High-speed varifocal imaging with a tunable acoustic gradient index of refraction lens. *Opt. Lett.* 33 2146–2148. 10.1364/OL.33.002146 18794959

[B76] Nadella K. M. N. S. Roš H. Baragli C. Griffiths V. A. Konstantinou G. Koimtzis T. (2016). Random access scanning microscopy for 3D imaging in awake behaving animals. *Nat. Methods* 13 1001–1004. 10.1038/nMeth.4033 27749836 PMC5769813

[B77] Nikolenko V. Poskanzer K. E. Yuste R. (2007). Two-photon photostimulation and imaging of neural circuits. *Nat. Methods* 4 943–950. 10.1038/NMETH1105 17965719

[B78] Nikolenko V. Watson B. O. Araya R. Woodruff A. Peterka D. S. Yuste R. (2008). SLM microscopy: scanless two-photon imaging and photostimulation with spatial light modulators. *Front. Neural Circuits* 2:5. 10.3389/neuro.04.005.2008 19129923 PMC2614319

[B79] Olarte O. E. Andilla J. Artigas D. Loza-Alvarez P. (2015). Decoupled illumination detection in light sheet microscopy for fast volumetric imaging. *Optica* 2 702–705. 10.1364/OPTICA.2.000702

[B80] Olivié G. Giguère D. Vidal F. Ozaki T. Kieffer J.-C. Nada O. (2008). Wavelength dependence of femtosecond laser ablation threshold of corneal stroma. *Opt. Express* 16 4121–4129. 10.1364/OE.16.004121 18542509

[B81] Oron D. Papagiakoumou E. Anselmi F. Emiliani V. (2012). Two-photon optogenetics. *Prog. Brain Res.* 196 119–143. 10.1016/B978-0-444-59426-6.00007-0 22341324

[B82] Oron D. Tal E. Silberberg Y. (2005). Scanningless depth-resolved microscopy. *Opt. Express* 13 1468–1476. 10.1364/OPEX.13.001468 19495022

[B83] Ozbay B. N. Futia G. L. Ma M. Bright V. M. Gopinath J. T. Hughes E. G. (2018). Three dimensional two-photon imaging of neuronal activity in freely moving mice using a miniature fiber coupled microscope with active axial-scanning. *bioRxiv* [Preprint]. 10.1101/226431 29802371 PMC5970169

[B84] Packer A. M. Peterka D. S. Hirtz J. J. Prakash R. Deisseroth K. Yuste R. (2012). Two-photon optogenetics of dendritic spines and neural circuits. *Nat. Methods* 9 1171–1179. 10.1038/nmeth.2249 23142873 PMC3518588

[B85] Packer A. M. Russell L. E. Dalgleish H. W. P. Häusser M. (2015). Simultaneous all-optical manipulation and recording of neural circuit activity with cellular resolution in vivo. *Nat. Methods* 12 140–146. 10.1038/nmeth.3217 25532138 PMC4933203

[B86] Papagiakoumou E. (2013). Optical developments for optogenetics. *Biol. Cell* 105 443–464. 10.1111/boc.201200087 23782010

[B87] Papagiakoumou E. Anselmi F. Bègue A. de Sars V. Glückstad J. Isacoff E. Y. (2010). Scanless two-photon excitation of channelrhodopsin-2. *Nat. Methods* 7 848–854. 10.1038/nmeth.1505 20852649 PMC7645960

[B88] Papagiakoumou E. Bègue A. Leshem B. Schwartz O. Stell B. M. Bradley J. (2013). Functional patterned multiphoton excitation deep inside scattering tissue. *Nat. Photonics* 7 274–278. 10.1038/nphoton.2013.9

[B89] Papagiakoumou E. de Sars V. Oron D. Emiliani V. (2008). Patterned two-photon illumination by spatiotemporal shaping of ultrashort pulses. *Opt. Express* 16 22039–22047. 10.1364/OE.16.022039 19104638

[B90] Papagiakoumou E. Ronzitti E. Chen I.-W. Gajowa M. Picot A. Emiliani V. (2018). Two-photon optogenetics by computer-generated holography. *Neuromethods* 133 175–197. 10.1007/978-1-4939-7417-7_10

[B91] Pégard N. Mardinly A. Oldenburg I. Waller L. Adesnik H. (2018). *Partially Coherent Holographic temporal focusing for 3D light sculpting with single neuron resolution. Opt. InfoBase Conf. Pap. Part F88-B*, 4–5. 10.1364/BRAIN.2018.BW2C.2∗

[B92] Pégard N. M. Oldenburg I. Sridharan S. Walller L. Adesnik H. (2017). 3D scanless holographic optogenetics with temporal focusing. *Nat. Commun.* 8:1228. 10.1038/s41467-017-01031-3 29089483 PMC5663714

[B93] Peron S. P. Freeman J. Iyer V. Guo C. Svoboda K. (2015). A cellular resolution map of barrel cortex activity during tactile behavior. *Neuron* 86 783–799. 10.1016/j.neuron.2015.03.027 25913859

[B94] Picot A. Dominguez S. Liu C. Chen I. W. Tanese D. Ronzitti E. (2018). Temperature rise under two-photon optogenetic brain stimulation. *Cell Rep.* 24 1243–1253.e5. 10.1016/j.celrep.2018.06.119 30067979

[B95] Piestun R. Spektor B. Shamir J. (1996). Wave fields in three dimensions: analysis and synthesis. *J. Opt. Soc. Am. A* 13 1837–1848. 10.1364/JOSAA.13.001837 8744164

[B96] Power R. M. Huisken J. (2017). A guide to light-sheet fluorescence microscopy for multiscale imaging. *Nat. Methods* 14 360–373. 10.1038/nmeth.4224 28362435

[B97] Prakash R. Yizhar O. Grewe B. Ramakrishnan C. Wang N. Goshen I. (2012). Two-photon optogenetic toolbox for fast inhibition, excitation and bistable modulation. *Nat. Methods* 9 1171–1179. 10.1038/nmeth.2215 23169303 PMC5734860

[B98] Prevedel R. Verhoef A. J. Pernía-Andrade A. J. Weisenburger S. Huang B. S. Nöbauer T. (2016). Fast volumetric calcium imaging across multiple cortical layers using sculpted light. *Nat. Methods* 13 1021–1028. 10.1038/nmeth.4040 27798612 PMC5531274

[B99] Prevedel R. Yoon Y.-G. Hoffmann M. Pak N. Wetzstein G. Kato S. (2014). Simultaneous whole-animal 3D imaging of neuronal activity using light-field microscopy. *Nat. Methods* 11 727–730. 10.1038/nmeth.2964 24836920 PMC4100252

[B100] Quirin S. Peterka D. S. Yuste R. (2013). Instantaneous three-dimensional sensing using spatial light modulator illumination with extended depth of field imaging. *Opt. Express* 21 16007–16021. 10.1364/OE.21.016007 23842387 PMC3971059

[B101] Quirin S. Vladimirov N. Yang C.-T. Peterka D. S. Yuste R. Ahrens M. B. (2016). Calcium imaging of neural circuits with extended depth-of-field light-sheet microscopy. *Opt. Lett.* 41 855–858. 10.1364/OL.41.000855 26974063 PMC4894304

[B102] Reddy G. D. Kelleher K. Fink R. Saggau P. (2008). Three-dimensional random access multiphoton microscopy for functional imaging of neuronal activity. *Nat. Neurosci.* 11 713–720. 10.1038/nn.2116 18432198 PMC2747788

[B103] Rickgauer J. P. Deisseroth K. Tank D. W. (2014). Simultaneous cellular-resolution optical perturbation and imaging of place cell firing fields. *Nat. Neurosci.* 17 1816–1824. 10.1038/nn.3866 25402854 PMC4459599

[B104] Rickgauer J. P. Tank D. W. (2009). Two-photon excitation of channelrhodopsin-2 at saturation. *Proc. Natl. Acad. Sci. U.S.A.* 106 15025–15030. 10.1073/pnas.0907084106 19706471 PMC2736443

[B105] Rodríguez C. Liang Y. Lu R. Ji N. (2018). Three-photon fluorescence microscopy with an axially elongated Bessel focus. *Opt. Lett.* 43 1914–1917. 10.1364/OL.43.001914 29652397 PMC5986555

[B106] Ronzitti E. Conti R. Zampini V. Tanese D. Foust A. J. Klapoetke N. (2017a). Sub-millisecond optogenetic control of neuronal firing with two-photon holographic photoactivation of Chronos. *J. Neurosci.* 37 10679–10689. 10.1523/JNEUROSCI.1246-17.2017 28972125 PMC5666587

[B107] Ronzitti E. Ventalon C. Canepari M. Forget B. C. Papagiakoumou E. Emiliani V. (2017b). Recent advances in patterned photostimulation for optogenetics. *J. Opt.* 19:113001 10.1088/2040-8986/aa8299

[B108] Rupprecht P. Prendergast A. Wyart C. Friedrich R. W. (2016). Remote z-scanning with a macroscopic voice coil motor for fast 3D multiphoton laser scanning microscopy. *Biomed. Opt. Express* 7 1656–1671. 10.1364/BOE.7.001656 27231612 PMC4871072

[B109] Seelig J. D. Jayaraman V. (2015). Neural dynamics for landmark orientation and angular path integration. *Nature* 521 186–191. 10.1038/nature14446 25971509 PMC4704792

[B110] Shemesh O. A. Tanese D. Zampini V. Linghu C. Piatkevich K. Ronzitti E. (2017). Temporally precise single-cell resolution optogenetics. *Nat. Neurosci.* 20 1796–1806. 10.1038/s41593-017-0018-8 29184208 PMC5726564

[B111] Sofroniew N. J. Flickinger D. King J. Svoboda K. (2016). A large field of view two-photon mesoscope with subcellular resolution for in vivo imaging. *eLife* 5:e14472. 10.7554/eLife.14472 27300105 PMC4951199

[B112] Stirman J. N. Smith I. T. Kudenov M. W. Smith S. L. (2016). Wide field-of-view, multi-region, two-photon imaging of neuronal activity in the mammalian brain. *Nat. Biotechnol.* 34 857–862. 10.1038/nbt.3594 27347754 PMC4980167

[B113] Sun B. Salter P. S. Roider C. Strauss J. Heberle J. Booth M. J. (2018). Four-dimensional light shaping?: manipulating ultrafast spatio- temporal foci in space and time. *Light Sci. Appl.* 7:17117 10.1038/lsa.2017.117 PMC6107044 30839626

[B114] Szabo V. Ventalon C. De Sars V. Bradley J. Emiliani V. (2014). Spatially selective holographic photoactivation and functional fluorescence imaging in freely behaving mice with a fiberscope. *Neuron* 84 1157–1169. 10.1016/j.neuron.2014.11.005 25433638

[B115] Tanese D. Weng J.-Y. Zampini V. De Sars V. Canepari M. Rozsa B. (2017). Imaging membrane potential changes from dendritic spines using computer-generated holography. *Neurophotonics* 4:031211. 10.1117/1.NPh.4.3.031211 28523281 PMC5428833

[B116] Tomer R. Khairy K. Amat F. Keller P. J. (2012). Quantitative high-speed imaging of entire developing embryos with simultaneous multiview light-sheet microscopy. *Nat. Methods* 9 755–763. 10.1038/nmeth.2062 22660741

[B117] Vogt N. (2015). All-optical electrophysiology in behaving animals. *Nat. Methods* 12:101. 10.1038/nmeth.3272 25798468

[B118] Yang S. Papagiakoumou E. Guillon M. de Sars V. Tang C. M. Emiliani V. (2011). Three-dimensional holographic photostimulation of the dendritic arbor. *J. Neural Eng.* 8:46002. 10.1088/1741-2560/8/4/046002 21623008

[B119] Yang W. Carrillo-reid L. Bando Y. Peterka D. S. Yuste R. (2018). Simultaneous two-photon optogenetics and imaging of cortical circuits in three dimensions. *eLife* 7:e32671. 10.7554/eLife.32671 29412138 PMC5832414

[B120] Yang W. Miller J. K. Carrillo-Reid L. Pnevmatikakis E. Paninski L. Yuste R. (2016). Simultaneous multi-plane imaging of neural circuits. *Neuron* 89 269–284. 10.1016/j.neuron.2015.12.012 26774159 PMC4724224

[B121] Yang W. Yuste R. (2017). In vivo imaging of neural activity. *Nat. Methods* 14 349–359. 10.1038/nmeth.4230 28362436 PMC5903578

[B122] Yang W. Yuste R. (2018). Holographic imaging and photostimulation of neural activity. *Curr. Opin. Neurobiol.* 50 211–221. 10.1016/j.conb.2018.03.006 29660600

[B123] Yizhar O. Fenno L. E. Davidson T. J. Mogri M. Deisseroth K. (2011). Optogenetics in neural systems. *Neuron* 71 9–34. 10.1016/j.neuron.2011.06.004 21745635

[B124] Zhu G. van Howe J. Durst M. Zipfel W. Xu C. (2005). Simultaneous spatial and temporal focusing of femtosecond pulses. *Opt. Express* 13 2153–2159. 10.1364/OPEX.13.002153 19495103

[B125] Zong W. Wu R. Li M. Hu Y. Li Y. Li J. (2017). Fast high-resolution miniature two-photon microscopy for brain imaging in freely behaving mice. *Nat. Methods* 14 713–719. 10.1038/nmeth.4305 28553965

[B126] Zou Y. Chau F. S. Zhou G. (2017). Ultra-compact optical zoom endoscope using solid tunable lenses. *Opt. Express* 25 20675–20688. 10.1364/OE.25.020675 29041746

